# Cuproptosis-driven reprogramming of fibroblast communication by GK is associated with the immune microenvironment in diabetic foot ulcers

**DOI:** 10.3389/fimmu.2025.1687806

**Published:** 2026-01-09

**Authors:** Tianbo Li, Jiangning Wang, Lei Gao

**Affiliations:** Department of Plastic Surgery, Capital Medical University Affiliated Beijing Shijitan Hospital, Beijing, China

**Keywords:** cell communication, cuproptosis, diabetic foot ulcers, fibroblast, GK, immune microenvironment, machine learning, single-cell RNA sequencing

## Abstract

**Background:**

Diabetic foot ulcers (DFUs) involve chronic inflammation and impaired healing. Emerging evidence implicates copper-dependent regulated cell death (cuproptosis) in immune microenvironment regulation, but its role in DFU fibroblast-immune crosstalk remains unknown.

**Methods:**

We analyzed scRNA-seq datasets (GSE231643, GSE134431) from DFU/healthy skin. After quality control (25,198 cells retained) and clustering, fibroblasts with elevated cuproptosis scores were identified via ssGSEA. Fibroblast-specific DEGs were screened (Wilcoxon rank-sum; |log_2_FC|>1, p<0.05) and functionally annotated (KEGG/GO). Machine learning (LASSO, SVM-RFE, Random Forest) pinpointed diagnostic biomarkers, validated by ROC analysis. Immune infiltration (CIBERSORT) and cellular communication (CellChat) were assessed in high/low biomarker groups. *In vivo* validation used nine SD rats (normal/wound/diabetic wound groups; n=3); GK mRNA/protein levels were measured via PCR/Western blot.

**Results:**

Through scRNA-seq analysis of 33,095 cells (25,198 post-quality control), fibroblasts exhibited the highest cuproptosis-related pathway scores. A total of 543 fibroblast-specific DEGs were identified, significantly enriched in pathways such as PI3K-Akt, chemokine signaling, NF-κB, IL-17 signaling, and AGE-RAGE signaling. GO analysis revealed enrichment in processes associated with extracellular matrix organization, inflammation regulation, and immune signaling. Integrated analyses involving bulk transcriptomics and machine learning identified GK as a robust cuproptosis-associated diagnostic biomarker (AUC=0.929, 95% CI: 0.714–1.000). GK expression positively correlated with neutrophil infiltration (R=0.95, p=0.00081) and negatively correlated with CD8^+^ T cells (R=−0.77, p=0.044) and follicular helper T cells (R=−0.89, p=0.0073). CellChat analysis demonstrated that high GK expression significantly enhanced fibroblast-mediated communication with immune cells via critical signaling pathways (MK, HGF, IGFBP, KIT), reshaping the immune-regulatory network within DFU tissues. Animal experiments further validated these findings: Quantitative PCR demonstrated that GK mRNA levels were significantly elevated in both wound model and diabetic wound model groups compared with the normal group, with the diabetic wound group showing significantly higher expression than the wound model. Consistent with mRNA findings, Western blot analysis revealed a progressive increase in GK protein expression across the normal, wound model, and diabetic wound model groups.

**Conclusions:**

Our data indicate that the cuproptosis-related gene GK is associated with alterations in DFU immune communication through fibroblast-centric networks, as reflected by correlations with immune infiltration and predicted ligand–receptor crosstalk. GK therefore represents a promising candidate diagnostic biomarker and a potential therapeutic target for DFUs that warrants further functional validation.

## 1 Introduction

Diabetic foot ulcers (DFUs) is one of the most debilitating and costly complications of diabetes mellitus, affecting up to 25% of patients during their lifetime and accounting for the majority of non-traumatic lower-limb amputations worldwide (1, 2). Despite advances in glucose control and standard wound care, overall healing rates remain unsatisfactory and recurrence is common, underscoring an urgent need for new mechanistic insights and targeted therapies (3, 4). Accumulating evidence indicates that non-healing in DFUs is driven by a persistent, dysregulated inflammatory milieu in which rapid‐responding innate immune cells predominate, adaptive immunity is impaired, and stromal-cell function is profoundly altered (5).

Among the stromal compartments, dermal fibroblasts play a dual role as producers of the extracellular matrix and as immunoregulatory “sentinels” that modulate leukocyte recruitment, phenotype, and effector functions through cytokines, chemokines, and direct ligand–receptor interactions (6). Single-cell RNA sequencing (scRNA-seq) has recently revealed marked heterogeneity within the fibroblast pool during cutaneous repair and identified discrete subpopulations with pro-inflammatory or pro-resolving programs (7). How these fibroblast subsets orchestrate cell-to-cell communication within the DFUs microenvironment, however, remains poorly defined.

A parallel line of investigation has uncovered cuproptosis, a newly described form of regulated cell death triggered by the intracellular accumulation of redox-active copper that targets lipoylated tricarboxylic-acid (TCA) cycle enzymes and disrupts mitochondrial metabolism (8, 9). Emerging data suggest that dysregulated copper homeostasis is intertwined with chronic inflammatory and metabolic diseases, including diabetic complications, and that cuproptosis-related gene signatures may predict disease activity (10). Yet, the functional consequences of cuproptosis signaling on stromal–immune crosstalk in chronic wounds have not been elucidated.

In this study, we integrated bulk transcriptomic and scRNA-seq datasets from DFUs and healthy skin to (i) quantify cuproptosis pathway activity across cell types, (ii) pinpoint fibroblast-enriched transcriptional programs associated with copper-driven stress, (iii) delineate changes in immune-cell infiltration patterns, and (iv) reconstruct ligand–receptor networks that underlie fibroblast-immune communication using CellPhoneDB and CellChat computational frameworks (11, 12). By coupling stringent differential-expression filtering with multiple machine-learning algorithms, we sought to identify and validate novel cuproptosis-linked molecular determinants that could reshape the immune landscape of DFUs and serve as promising diagnostic or therapeutic targets.

## 2 Materials and methods

### 2.1 Data acquisition and single-cell RNA sequencing preprocessing

Single-cell RNA sequencing datasets were retrieved from the Gene Expression Omnibus (GEO) database (accession number: GSE231643) (13). These data included skin tissues from diabetic foot ulcers (DFUs) patients (DFUs 1–3) and healthy controls (Healthy 1–5). Data processing and subsequent analyses were performed using the Seurat R package (v4.0.6). Firstly, strict quality control was applied based on gene counts per cell (nFeature_RNA), total RNA counts per cell (nCount_RNA), and mitochondrial gene percentage (percent.MT).

1. nFeature_RNA (number of genes detected per cell), reflecting library complexity;
2. nCount_RNA (total UMI counts per cell), representing sequencing depth; and
3. percent.MT (percentage of mitochondrial transcripts), indicating potential cellular stress or damage.

Low-quality cells were excluded using thresholds of nFeature_RNA < 8,000, nCount_RNA < 30,000, and percent.MT < 15%.

After quality control, cells underwent log normalization, identification of highly variable genes, principal component analysis (PCA), and UMAP dimensionality reduction. Batch effects across samples were corrected using the Harmony algorithm with default parameter settings, specifying orig.ident as the batch variable. The corrected embeddings were then used for graph-based clustering: FindNeighbors was performed using the first 30 principal components, followed by Louvain clustering (FindClusters), with the resolution chosen empirically to achieve stable and biologically interpretable major cell groups.

No dedicated doublet-removal tool (e.g., DoubletFinder or Scrublet) was applied; instead, potential multiplets were indirectly mitigated by the upper-bound nFeature_RNA/nCount_RNA filters and by marker-based inspection of cluster purity.

Cell clusters were annotated by cross-referencing canonical marker genes and the CellMarker database, resulting in identification of major cell types including fibroblasts, macrophages, T cells, B cells, dendritic cells, endothelial cells, granulocytes, and mast cells.

Additionally, bulk RNA-seq data from GEO dataset GSE134431 (containing six normal and seven DFUs samples) were downloaded to validate scRNA-seq results (14).

### 2.2 Identification of target fibroblast subpopulation driven by cuproptosis scores and functional enrichment analyses

Single-sample gene set enrichment analysis (ssGSEA) was conducted using previously reported cuproptosis-related gene sets to quantify cuproptosis pathway activity across cell populations. Fibroblasts, identified as exhibiting the highest cuproptosis scores, were selected for further analysis.

To delineate fibroblast-specific transcriptional signatures, differential expression analysis was performed using the Wilcoxon rank-sum test. Fibroblasts were compared against all other cell types (T cells, B cells, macrophages, dendritic cells, granulocytes, mast cells, and endothelial cells). Differentially expressed genes (DEGs) were identified based on an absolute log2 fold-change >1 and a p-value <0.05. A total of 543 fibroblast-specific DEGs were obtained and further subjected to functional annotation analyses. Kyoto Encyclopedia of Genes and Genomes (KEGG) pathway enrichment analysis and Gene Ontology (GO) analysis, encompassing biological processes (BP), cellular components (CC), and molecular functions (MF), were conducted. Enrichment results were visualized by fold enrichment and −log10(p-value).

### 2.3 Screening of diagnostic genes using machine learning approaches

To screen for potential cuproptosis-associated fibroblast diagnostic genes in DFUs, we intersected the DEGs derived from fibroblasts (Fib_degs) with those identified at the bulk transcriptomic level (DFUs_degs), resulting in 67 common candidate genes. Subsequently, we employed three robust machine learning algorithms—least absolute shrinkage and selection operator (LASSO) regression, support vector machine-recursive feature elimination (SVM-RFE), and random forest (RF)—to select the most informative and predictive genes. Analyses were performed using R packages including “glmnet”, “kernlab”, and “randomForest”. A common intersection gene identified by all three algorithms was designated as the critical diagnostic biomarker. The receiver operating characteristic (ROC) curve was generated using the “pROC” R package to validate the predictive accuracy and reliability of the selected biomarker gene.

For LASSO, the regularization parameter (λ) was selected via internal cross-validation as implemented in the glmnet workflow, and features with non-zero coefficients at the selected λ (minimum cross-validated error or the 1-SE rule for a more parsimonious model) were retained. For SVM-RFE, features were recursively eliminated and model performance was evaluated by cross-validation at each step; the feature subset with the lowest cross-validated prediction error (RMSE) was selected. For RF, candidate genes were ranked by variable importance (mean decrease in Gini) as a complementary feature-selection measure. Because this stage aimed at consensus biomarker discovery rather than optimization of a final multi-feature classifier, we did not implement nested cross-validation; instead, the final biomarker was defined as the gene consistently identified by all three algorithms. Diagnostic performance metrics (AUC and 95% CI) were derived from ROC analysis using the “pROC” package on the bulk transcriptomic validation dataset (GSE134431).

### 2.4 Immune cell infiltration estimation

To comprehensively characterize the immune microenvironment associated with diabetic foot ulcers (DFUs) and explore its potential relationship with cuproptosis-related genes, immune cell infiltration profiles were quantitatively analyzed using the CIBERSORT algorithm. CIBERSORT is a deconvolution method based on support vector regression (SVR) that accurately infers the relative proportions of 22 immune cell subsets within complex tissue samples from transcriptomic data. Specifically, the normalized bulk RNA-sequencing expression matrices from control (healthy) and DFU groups were uploaded to CIBERSORT, utilizing the LM22 signature matrix as a reference. This allowed for detailed quantification of various immune cell types, including T cells, B cells, macrophages, neutrophils, natural killer (NK) cells, and dendritic cells, among others.

CIBERSORT analysis was performed on all bulk RNA-seq samples from GSE134431, including six normal controls and seven DFUs (total n = 13). Group comparisons across the inferred immune cell types were conducted using the Wilcoxon rank-sum test, and multiple comparisons were corrected using the false discovery rate (FDR) to control for type I error across cell-type comparisons.

Subsequently, differences in immune cell compositions between control and DFUs samples were statistically assessed, and immune-cell interactions were investigated through correlation analyses, revealing potential cooperative or antagonistic relationships among immune subpopulations. Furthermore, correlation analyses were conducted to examine the relationship between expression levels of candidate cuproptosis-related genes (identified through previous analyses) and immune cell infiltration abundances. Pearson correlation coefficients and corresponding p-values were calculated to identify significant associations. This integrative analytical approach provided insights into how cuproptosis-related molecular events might influence immune cell recruitment, functional polarization, and the overall immune landscape remodeling observed in DFU tissues.

### 2.5 Cell–cell communication analysis

To investigate how cuproptosis-related pathways influence cell–cell communication within the diabetic foot ulcer (DFU) microenvironment, we performed a comprehensive reconstruction and comparative analysis of cellular communication networks using the CellChat package (v1.6.1). All cells obtained from scRNA-seq data were divided into two distinct groups based on median expression levels of candidate cuproptosis-related genes identified from the previous differential expression and machine learning analyses. Accordingly, two separate Seurat objects representing high-expression and low-expression cell populations were constructed, and normalized expression matrices were extracted.

Subsequently, we identified overexpressed ligands and receptors, as well as enriched ligand–receptor interaction pairs in each group, using the identifyOverExpressedGenes and identifyOverExpressedInteractions functions provided by CellChat. Communication probability between cell populations was calculated using computeCommunProb, and low-confidence interactions were filtered with filterCommunication. Pathway-level communication analyses were carried out using computeCommunProbPathway, and signaling similarities between cellular pairs were evaluated through computeNetSimilarity. The global communication networks and specific signaling pathways were visualized with CellChat’s built-in visualization tools, including netVisual_circle and netVisual_aggregate.

Finally, we employed the rankNet function to quantify and compare the contributions of different cell populations to communication networks between high- and low-expression groups. Emphasis was placed on signaling pathways closely associated with immune regulation, inflammation, and tissue repair processes, specifically MK, HGF, IGFBP, and KIT, to elucidate how cuproptosis-related molecular events might reshape the immune landscape and cellular communication dynamics in DFUs.

### 2.6 Animal experiments to validate GK expression associated with cuproptosis in diabetic wound models

#### 2.6.1 Animal selection and diabetic wound model establishment

Male Sprague-Dawley (SD) rats, aged 8–12 weeks, were employed to establish a diabetic wound model. Diabetes mellitus was induced using intraperitoneal injections of streptozotocin (STZ; 60 mg/kg body weight) administered once daily for five consecutive days. Blood glucose levels were monitored 1–2 weeks post-STZ injection, and rats exhibiting stable hyperglycemia (blood glucose ≥16.7 mmol/L) were considered diabetic and included in subsequent experiments. A chronic diabetic foot ulcer (DFU)-like wound model was created by inducing a full-thickness skin defect on the rat dorsal area. Briefly, after confirming diabetic status, a standardized circular full-thickness skin wound (6 mm in diameter) was surgically established on the dorsal region under sterile conditions to simulate chronic DFUs.

#### 2.6.2 Experimental grouping and tissue sampling

A total of nine SD rats were randomly divided into three groups (n=3 per group): normal control group—rats received no treatment, with normal skin tissue collected; wound model group—rats underwent skin wounding procedures as described above; diabetic wound model group—rats first underwent STZ-induced diabetes followed by the skin wound procedure, representing chronic diabetic wounds.

At designated time points post-wounding, tissues from the wound area (or corresponding normal skin for control rats) were collected, rapidly frozen in liquid nitrogen, and stored at −80°C until subsequent analyses.

#### 2.6.3 RNA extraction and quantitative real-time PCR

Total RNA was extracted from rat skin tissue samples using TRIzol reagent (Invitrogen, Carlsbad, CA, USA) following the manufacturer’s protocol. Extracted RNA was treated with DNase I (Thermo Scientific, USA) to remove genomic DNA contamination. Subsequently, cDNA was synthesized using the PrimeScript™ RT kit (Takara Bio, Shiga, Japan).

Quantitative PCR was performed using SYBR Green qPCR Master Mix (Bio-Rad, Hercules, CA, USA) on a Bio-Rad Real-Time PCR system. The reaction volume was 20 µL, with the housekeeping gene Gapdh as an internal control. PCR cycling conditions included an initial denaturation at 95°C for 5 min, followed by 40 cycles at 95°C for 15 s and 60°C for 30 s. Relative gene expression was calculated using the 2^−ΔΔCt method. Primer sequences were as follows:

Gapdh (internal control):
Forward: GGAGAGTGTTTCCTCGTCCC
Reverse: GATGGGCTTCCCGTTGATGA
Product length: 245 bp
Gk:
Forward: GAGGAATCCATGGGGGTGTC
Reverse: CCCCAGCTTTCATTAGGCCA
Product length: 177 bp

Data were presented as mean ± SEM, and statistical differences between groups were analyzed using one-way ANOVA followed by Tukey’s *post hoc* test.

#### 2.6.4 Protein extraction and Western blot analysis

Frozen tissue samples (50 mg) were homogenized in ice-cold RIPA lysis buffer supplemented with 1 mM phenylmethanesulfonyl fluoride (PMSF). Protein lysates were centrifuged at 12,000×g for 15 min at 4°C, and the protein concentration was determined using the BCA assay kit (Thermo Scientific, USA).

Subsequently, equal amounts of protein (30 μg per sample) were separated by 12% SDS-polyacrylamide gel electrophoresis (SDS-PAGE) and transferred onto PVDF membranes under a constant current of 200 mA for 90 min. Membranes were blocked with 5% non-fat dry milk in TBST for 1 h at room temperature. The membranes were then incubated overnight at 4°C with primary antibodies: anti-GK rabbit antibody (1:1,000 dilution, Abcam or specified manufacturer) and anti-GAPDH rabbit antibody (1:5,000 dilution, Proteintech or specified manufacturer). After washing, membranes were incubated with HRP-conjugated secondary antibodies (1:5,000 dilution; Proteintech, USA) for 1 h at room temperature.

Protein bands were visualized using enhanced chemiluminescence (ECL, Thermo Scientific, USA). Band intensities were quantified using ImageJ software, and relative protein expression was normalized against GAPDH expression. Data were expressed as mean ± SEM. Statistical differences among groups were evaluated using one-way ANOVA followed by Tukey’s *post hoc* test.

## 3 Results

### 3.1 Quality control, integration, and cell-type annotation of single-cell transcriptomic data

**Figure 1:**
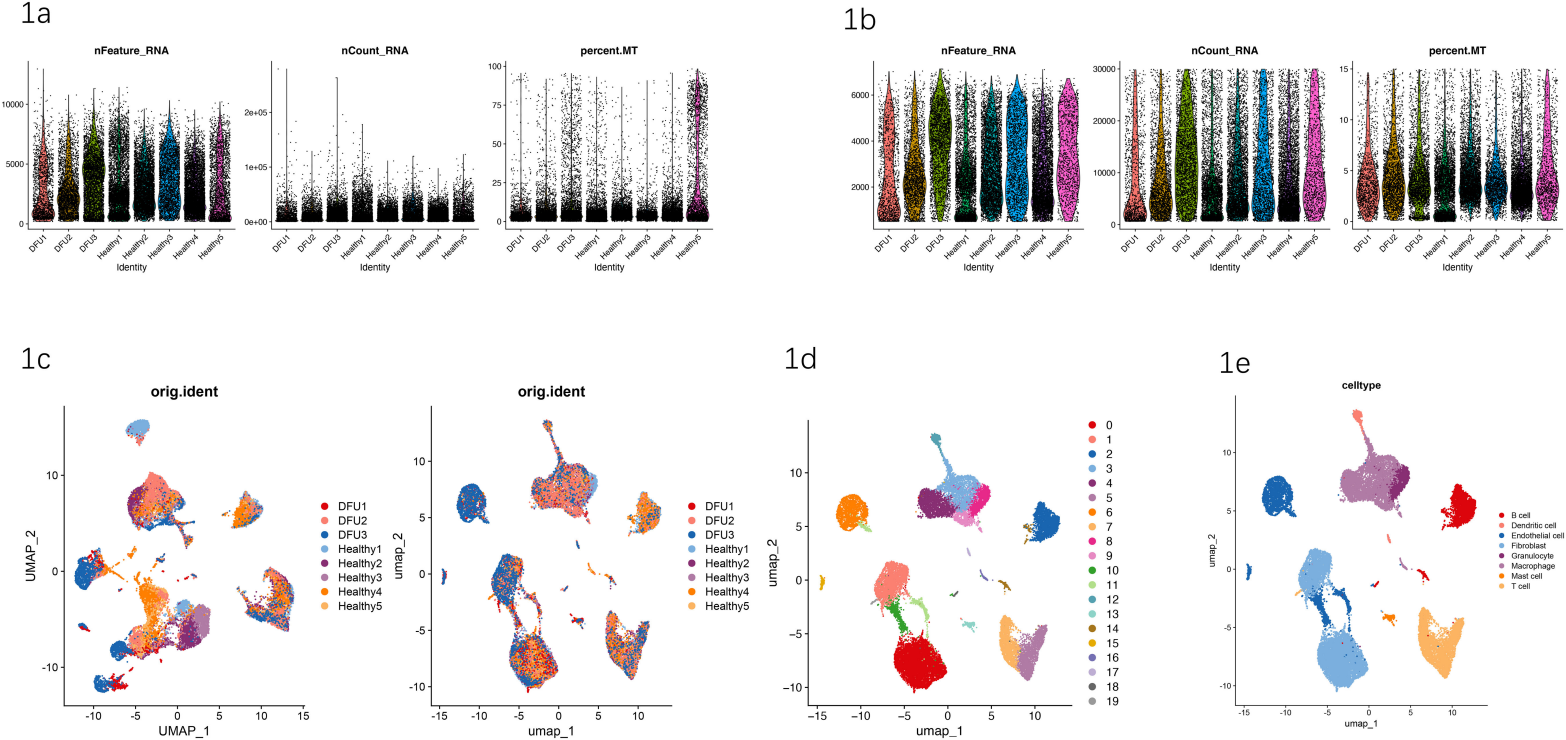
**(a)** Quality metrics of original single-cell RNA sequencing data. Distribution of the number of genes expressed per cell (nFeature_RNA), total RNA counts per cell (nCount_RNA), and mitochondrial gene percentage (percent.MT) across DFUs (DFUs 1–3) and healthy control samples (Healthy1–5) prior to quality control filtering. These QC distributions are provided for descriptive assessment of raw data quality and were not statistically tested. **(b)** Quality metrics after stringent quality control filtering. Violin plots illustrating distributions of nFeature_RNA, nCount_RNA, and percent.MT in retained high-quality cells from DFUs and healthy control samples, demonstrating that post-filtering metrics fell within acceptable ranges. **(c)** UMAP visualization by sample origin (orig.ident). Left: UMAP of the merged dataset before batch correction (colored by DFU1–3 and Healthy1–5). Right: UMAP after Harmony-based batch correction/integration, demonstrating improved mixing of cells across samples and minimal residual batch effects. **(d)** Identification of cell clusters in integrated single-cell transcriptome data. UMAP visualization depicting 20 distinct cell clusters identified by Louvain clustering, revealing marked cellular heterogeneity. **(e)** Cell-type annotation based on canonical markers. UMAP visualization displaying annotation results for eight major cell types (B cells, dendritic cells, endothelial cells, fibroblasts, granulocytes, macrophages, mast cells, and T cells) identified across integrated DFUs and healthy control samples.

Initially, 33,095 cells from diabetic foot ulcers (DFUs) and healthy control skin samples were subjected to rigorous quality control based on gene expression thresholds (≥3 cells per gene and ≥250 genes per cell). Original data distributions demonstrated substantial variation across samples, with notable differences in the number of genes expressed per cell (nFeature_RNA), total RNA transcripts per cell (nCount_RNA), and mitochondrial gene percentage (percent.MT). QC metrics (nFeature_RNA, nCount_RNA, and percent.MT) showed sample-to-sample variability. The differences observed in DFU versus healthy samples in **Figure 1a** are presented descriptively and were not subjected to hypothesis testing, as these measures primarily reflect technical QC rather than biological endpoints. After applying stringent filtering criteria (nFeature_RNA < 8,000, nCount_RNA < 30,000, and percent.MT < 15%), 25,198 high-quality cells remained for subsequent analyses, with all quality metrics consistently within acceptable ranges (**Supplementary Table S1**)(**Figure 1b**).

Subsequently, Uniform Manifold Approximation and Projection (UMAP) was used to visualize the integrated dataset. The UMAP visualization revealed effective data integration and minimal batch effects among DFUs and healthy samples, although some subtle sample-specific cell distribution differences were still discernible (**Figure 1c**). Louvain clustering analysis identified 20 distinct cell clusters, highlighting significant cellular heterogeneity across all samples (**Figure 1d**). Each cell cluster was further annotated using canonical markers and known gene-expression profiles, resulting in the identification of eight primary cell types: macrophages, fibroblasts, T cells, B cells, dendritic cells, endothelial cells, granulocytes, and mast cells (**Figure 1e**). These clearly annotated cellular populations provided an essential framework for exploring cell-specific roles in DFU pathogenesis.

### 3.2 Identification and functional enrichment analysis of fibroblast-specific differentially expressed genes

**Figure 2:**
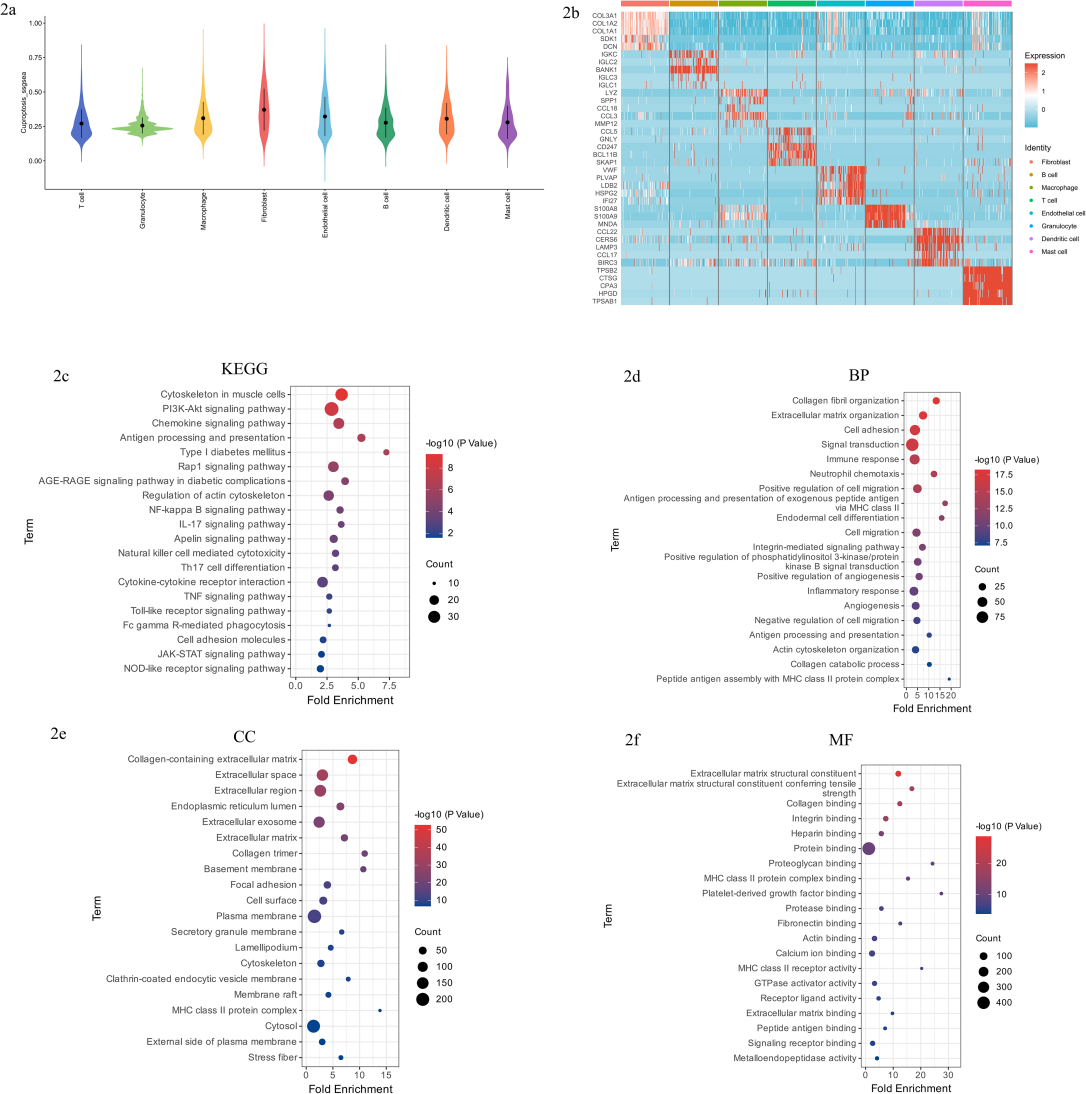
**(a)** Cuproptosis enrichment scores across cell types. Bar chart illustrating significantly higher ssGSEA scores for cuproptosis-associated pathways in fibroblasts compared with other cell populations. **(b)** Heatmap of representative fibroblast-specific DEGs. Heatmap displaying expression patterns of the top five DEGs (ranked by absolute logFC) across fibroblasts and other major cell types, highlighting fibroblast-specific expression signatures. **(c)** KEGG pathway enrichment analysis of fibroblast-specific DEGs. Dot plot illustrating significant enrichment in immune and metabolic pathways relevant to chronic inflammatory conditions, such as PI3K-Akt and NF-κB signaling. **(d)** GO biological process enrichment analysis of fibroblast-specific DEGs. Dot plot depicting significantly enriched biological processes, including collagen fibril organization, extracellular matrix organization, and immune regulation processes. **(e)** GO cellular component enrichment analysis of fibroblast-specific DEGs. Dot plot highlighting enrichment of DEGs in extracellular matrix structures, basement membrane, and focal adhesion, emphasizing fibroblast involvement in extracellular structural integrity. **(f)** GO molecular function enrichment analysis of fibroblast-specific DEGs. Dot plot demonstrating DEGs’ enrichment in molecular functions such as collagen binding, integrin binding, and extracellular matrix structural components, underpinning fibroblast’s multifunctional roles in DFUs.

To explore the potential role of fibroblasts in cuproptosis-associated pathways within diabetic foot ulcer (DFU) tissues, we first evaluated the enrichment scores of cuproptosis-related pathways across all major cell types using single-sample gene set enrichment analysis (ssGSEA). Notably, fibroblasts exhibited significantly higher cuproptosis enrichment scores compared to other cell populations (**Figure 2a**), indicating that fibroblasts exhibit the highest cuproptosis-related enrichment/signature among the major cell populations and were therefore prioritized for downstream analyses.

To further investigate the transcriptional characteristics unique to fibroblasts, we performed differential gene expression analysis comparing fibroblasts with other major cell types. This analysis resulted in the identification of 543 fibroblast-specific DEGs. Among these genes, the top five genes ranked by absolute log fold-change (logFC) exhibited remarkably high expression specificity in fibroblasts, clearly distinguishing this cell type from others (**Figure 2b**).

KEGG pathway enrichment analysis of the identified DEGs revealed significant associations with pathways related to immune regulation and metabolism, including the PI3K-Akt signaling pathway, chemokine signaling pathway, antigen processing and presentation, AGE-RAGE signaling pathway in diabetic complications, NF-κB signaling pathway, and IL-17 signaling pathway (**Figure 2c**). These pathways are broadly involved in cellular survival, immune activation, inflammation, and oxidative stress regulation, highlighting their potential roles in sustaining chronic inflammation characteristic of DFUs.

Gene Ontology (GO) enrichment analysis of the biological processes (BP) indicated significant involvement of these DEGs in immune response, tissue remodeling, and cell migration, notably collagen fibril organization, extracellular matrix organization, positive regulation of cell migration, inflammatory response, and signal transduction (**Figure 2d**). This underscores the dual functional capability of fibroblasts in both inflammatory signaling and tissue repair processes within DFUs lesions.

Analysis of cellular components (CC) revealed that these DEGs were predominantly enriched in extracellular structures such as the collagen-containing extracellular matrix, extracellular space, basement membrane, and focal adhesion (**Figure 2e**), suggesting a critical role in extracellular matrix integrity and cell–matrix interactions.

Furthermore, molecular function (MF) enrichment indicated robust associations with functions like extracellular matrix structural constituents, collagen binding, integrin binding, heparin binding, and MHC class II receptor activity (**Figure 2f**). These molecular functions form a coherent foundation for fibroblasts’ roles in matrix remodeling, cellular attachment, and immune antigen presentation in DFUs.

### 3.3 Identification of cuproptosis-associated diagnostic biomarker via machine learning approaches

**Figure 3:**
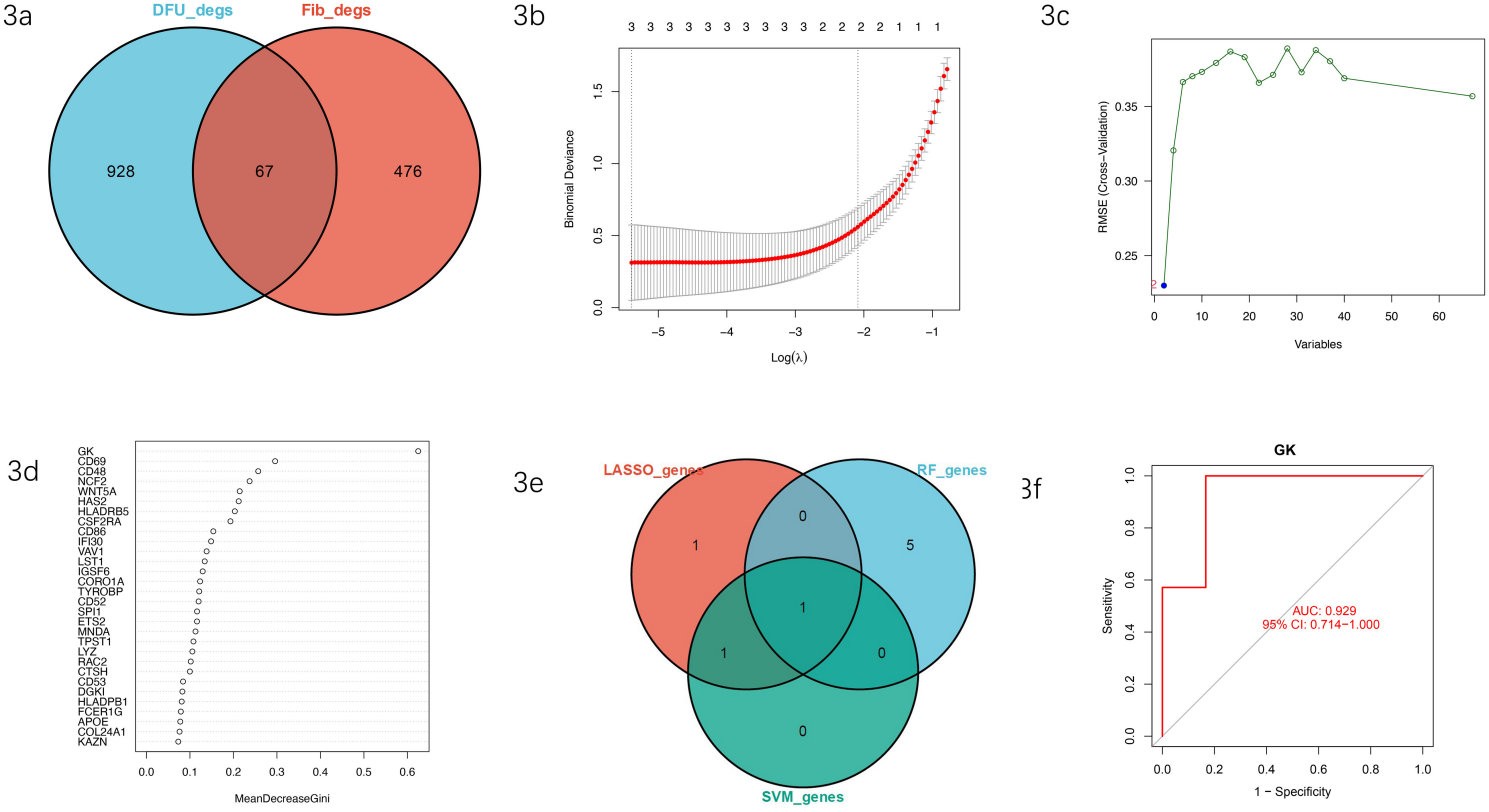
**(a)** Intersection of fibroblast-specific DEGs and bulk RNA-seq DEGs. Venn diagram depicting the intersection of fibroblast-specific cuproptosis-related DEGs (Fib_degs, n=476) and bulk RNA-seq-derived DEGs from DFU tissues (DFUs_degs, n=928), resulting in 67 candidate genes. **(b)** Feature selection by LASSO regression analysis. LASSO regression plot illustrating the binomial deviance versus log(λ), showing selection of three stable predictive genes. **(c)** Optimal gene subset identification by SVM-RFE analysis. Line graph representing the RMSE cross-validation error curve, indicating the optimal number of variables selected by SVM-RFE. **(d)** Variable importance ranking by random forest. Bar chart presenting genes ranked by mean decrease in Gini importance values, highlighting multiple significant candidate genes. **(e)** Intersection of candidate genes from three machine-learning algorithms. Venn diagram illustrating the overlap of selected biomarkers among LASSO, SVM-RFE, and random forest analyses, identifying GK as the sole intersecting biomarker. **(f)** ROC curve analysis for the diagnostic performance of GK. ROC curve depicting high sensitivity and specificity of GK in distinguishing DFUs from control samples, with an area under the curve (AUC) value of 0.929 (95% CI: 0.714–1.000).

To identify candidate biomarkers significantly associated with the cuproptosis pathway and specifically expressed in diabetic foot ulcers (DFUs) tissues, we initially intersected fibroblast-specific cuproptosis-associated DEGs (Fib_degs) with DEGs identified from bulk RNA-seq analyses of DFU samples (DFUs_degs). This intersection identified 67 overlapping genes, indicating their robust expression differences at both single-cell and bulk levels (**Figure 3a**).

Subsequently, these 67 genes were subjected to further feature selection using three robust machine-learning methods. LASSO regression analysis minimized binomial deviance by introducing an optimal regularization parameter (log λ), ultimately identifying three variables with stable predictive power (**Figure 3b**). The support vector machine-recursive feature elimination (SVM-RFE) approach identified an optimal subset of features based on minimal root mean square error (RMSE), highlighting a set of key variables with high classification accuracy (**Figure 3c**). Additionally, random forest (RF) analysis ranked genes by mean decrease in Gini importance, identifying multiple candidate genes with significant predictive contributions (**Figure 3d**).

Intersection of the candidate genes selected by these three independent machine-learning methods revealed that GK was the only common gene identified consistently, underscoring its robustness and potential as a critical biomarker (**Figure 3e**).

Finally, we conducted receiver operating characteristic (ROC) analysis to evaluate the diagnostic performance of GK. ROC curve analysis demonstrated excellent discriminatory power between DFUs and control tissues (AUC=0.929, 95% CI: 0.714–1.000), supporting GK’s potential utility as a diagnostic biomarker for DFUs linked to cuproptosis (**Figure 3f**). The ROC-based performance metrics reported here were derived from the bulk RNA-seq cohort used for model evaluation (via pROC), whereas the single-cell dataset and *in vivo* experiments were used as orthogonal biological validation rather than as an independent test set for AUC estimation.

Collectively, our multilayered selection approach successfully pinpointed GK as a stable, highly predictive, and cuproptosis-associated diagnostic biomarker, warranting further mechanistic exploration and validation.

### 3.4 Immune cell infiltration analysis via CIBERSORT and association with GK expression

**Figure 4:**
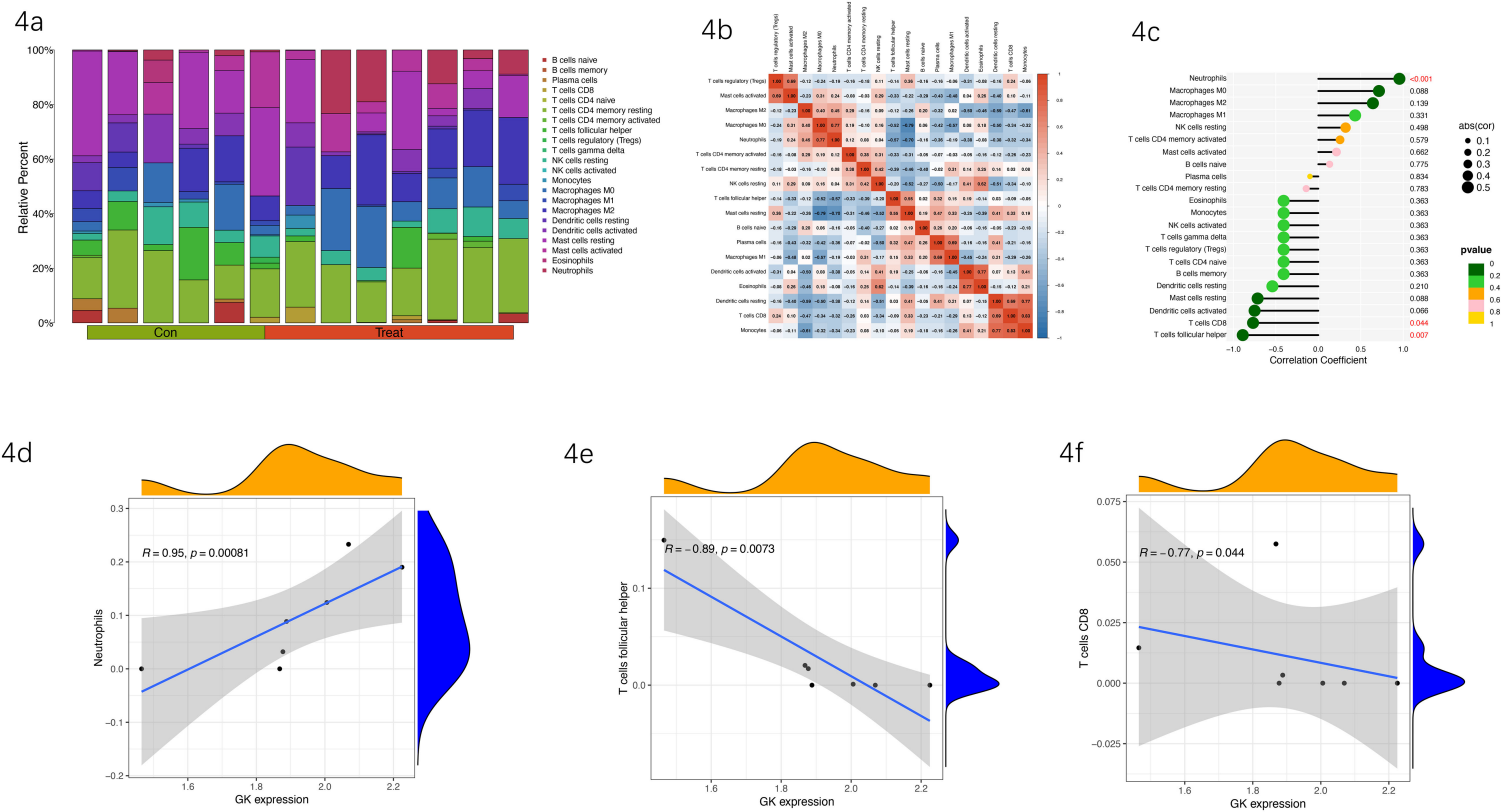
**(a)** Composition of immune cell infiltrates in DFU and control tissues. Stacked bar chart illustrating the relative proportions of 22 immune cell subsets, revealing marked differences in immune cell compositions between control (Con) and DFU (Treat) groups. **(b)** Correlation heatmap of immune cell subsets. Heatmap showing pairwise correlation coefficients among immune cell types, highlighting significant positive or negative interactions, particularly involving neutrophils, T cells, and macrophages. **(c)** Correlation analysis between GK expression and immune cell abundances. Scatter plot summarizing correlation coefficients and p-values between GK expression and various immune cell subsets, identifying neutrophils, follicular helper T cells, and CD8^+^ T cells as significantly correlated populations. **(d)** Positive correlation between GK expression and neutrophil infiltration. Scatter plot illustrating the significant positive association (R = 0.95, p = 0.00081) between GK expression levels and neutrophil abundance, showing a significant positive association between GK expression and neutrophil abundance. **(e)** Negative correlation between GK expression and follicular helper T-cell infiltration. Scatter plot displaying the significant negative correlation (R = −0.89, p = 0.0073) between GK expression levels and follicular helper T-cell infiltration, showing a significant negative association between GK expression and follicular helper T-cell abundance. **(f)** Negative correlation between GK expression and CD8^+^ T-cell infiltration. Scatter plot showing a significant negative correlation (R = −0.77, p = 0.044) between GK expression and CD8^+^ T-cell abundance, further showing a significant negative association between GK expression and CD8+ T-cell abundance.

To explore the potential role of the cuproptosis-related key gene GK within the immune microenvironment of diabetic foot ulcers (DFUs), we employed the CIBERSORT algorithm to quantitatively evaluate immune cell infiltration profiles. The relative abundances of 22 distinct immune cell subpopulations significantly differed between control and DFU groups, suggesting notable remodeling of the immune microenvironment during DFU progression (**Figure 4a**).

Further correlation analysis revealed complex interactive relationships between immune cell subsets. Specifically, strong correlations (both synergistic and antagonistic) were observed among neutrophils, T cells, macrophages, and other immune cells, reflecting an interactive and dynamic immune landscape within DFU tissues (**Figure 4b**).

Correlation analysis between GK expression and immune cell infiltration showed significant associations with several immune subsets (**Figure 4c**). Notably, GK expression exhibited a strong positive correlation with neutrophil infiltration (R = 0.95, p = 0.00081), suggesting its potential involvement in neutrophil-mediated inflammatory responses in DFUs (**Figure 4d**). Conversely, GK expression showed significant negative correlations with infiltration of follicular helper T cells (R = –0.89, p = 0.0073) and CD8^+^ T cells (R = –0.77, p = 0.044), suggesting a possible role for GK in modulating adaptive immune suppression or immune escape mechanisms (**Figures 4e, f**).

Taken together, these results indicate that GK is closely associated with immune cell infiltration profiles and may be involved in immune cell recruitment and functional states in DFUs. However, these associations are correlative and do not directly demonstrate that GK modulates immune cells. Thus, GK should currently be regarded as a cuproptosis-related gene linked to immune remodeling in DFUs, rather than a proven immunomodulatory driver. These findings support that GK expression is associated with distinct immune infiltration patterns in DFU tissues. However, causality cannot be inferred from these analyses.

### 3.5 Cell communication network remodeling associated with GK expression in the DFU microenvironment

To comprehensively understand the role of the cuproptosis-associated gene GK in reshaping cell communication within the diabetic foot ulcer (DFU) microenvironment, we analyzed cell–cell interactions using CellChat analysis. Cells from DFU tissues were stratified into GK-high and GK-low groups based on median GK expression, and interaction networks were reconstructed to identify potential signaling changes.

**Figure 5:**
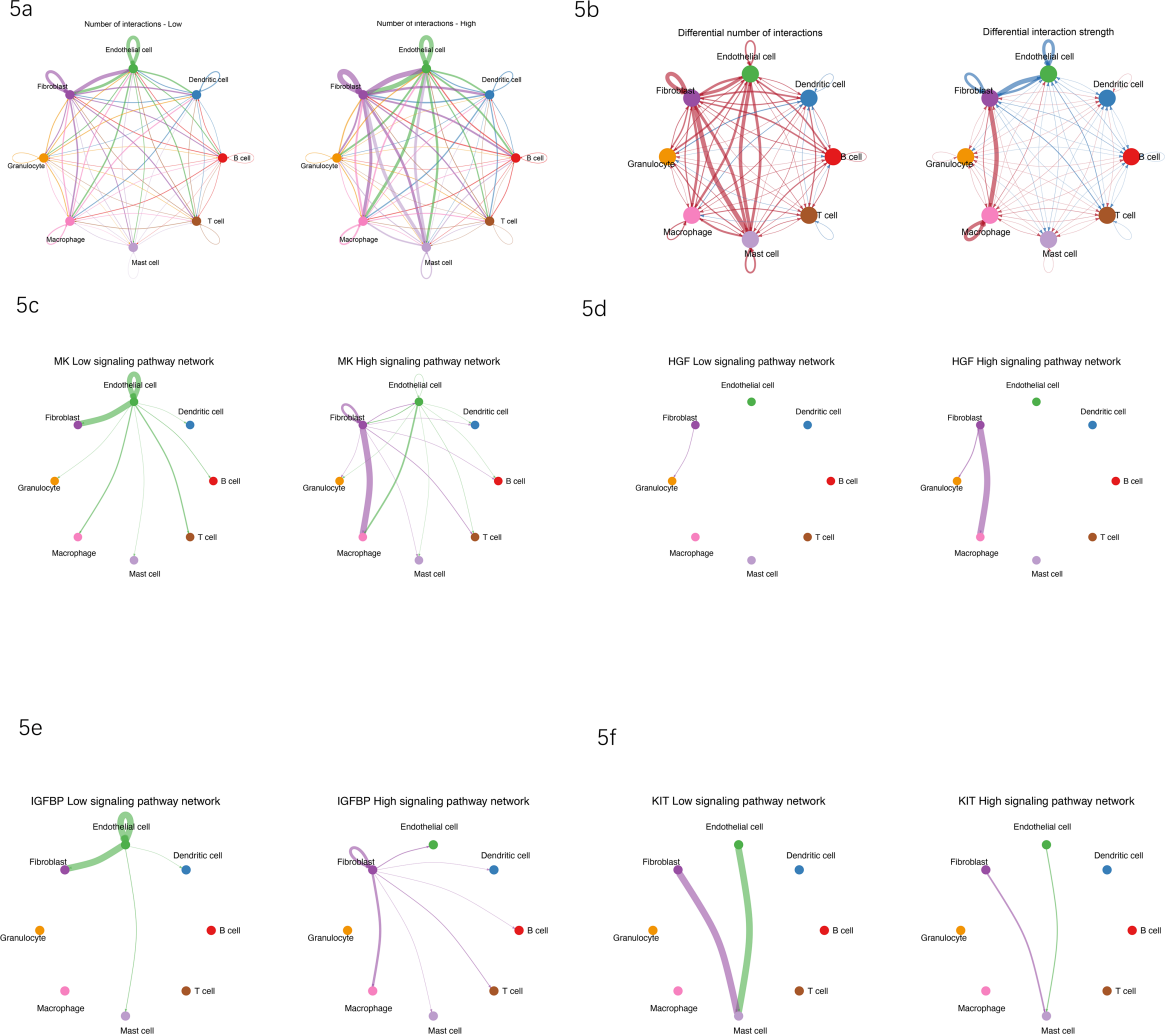
Reconstruction of cell–cell communication networks reveals significant remodeling under GK-high expression conditions in the DFU microenvironment. **(a)** Comparison of overall cellular interaction numbers between GK-low (left) and GK-high (right) groups. Increased density and complexity of cell–cell interactions were observed under high GK expression. **(b)** Differential analysis of cell–cell interaction networks between GK-low and GK-high groups. Left panel shows differential number of interactions; right panel shows differential interaction strength. Red lines indicate interactions increased in GK-high; blue lines indicate interactions decreased. **(c)** MK signaling pathway network remodeling under GK-low (left) and GK-high (right) conditions. Fibroblast-to-macrophage signaling significantly enhanced under GK-high condition, establishing fibroblasts as key signaling sources. **(d)** HGF signaling pathway network remodeling under GK-low (left) and GK-high (right) conditions. Fibroblast-originated signaling toward macrophages and granulocytes substantially increased under GK-high conditions. **(e)** IGFBP signaling pathway network remodeling under GK-low (left) and GK-high (right) conditions. GK-high fibroblasts exhibit expanded communication with immune cells (macrophages, granulocytes, and T cells), compared with limited endothelial-centric signaling in GK-low conditions. **(f)** KIT signaling pathway network remodeling under GK-low (left) and GK-high (right) conditions. GK-high fibroblasts transitioned from responsive to predominant regulatory cells, actively communicating signals to macrophages and mast cells, contrasting with endothelial-driven signaling in GK-low conditions. Note: The thickness of lines represents the intensity of cellular interactions, and arrows indicate the direction of ligand–receptor signaling. Enhanced fibroblast-mediated signaling under GK-high conditions highlights that higher GK expression is associated with stronger predicted fibroblast-mediated signaling in DFU tissues.

Analysis revealed significantly enhanced interaction frequency and strength among cells in the GK-high group compared with the GK-low group (**Figure 5a**). Specifically, fibroblasts, endothelial cells, macrophages, and granulocytes formed denser and more complex interaction networks under high GK expression conditions. Differential analyses further confirmed marked increases in both the number and strength of cell–cell interactions between fibroblasts and immune cells (macrophages, granulocytes, and mast cells) in the GK-high group, suggesting that higher GK expression is associated with increased predicted fibroblast–immune communication (CellChat inference) in DFUs (**Figure 5b**).

Detailed examination of specific signaling pathways revealed substantial changes associated with GK expression status. In the MK signaling pathway, fibroblasts strongly enhanced their signaling toward macrophages in the GK-high group, establishing fibroblasts as the central signaling source, whereas these interactions were substantially weaker in the GK-low group (**Figure 5c**). Similarly, the HGF signaling pathway exhibited intensified fibroblast-to-macrophage and fibroblast-to-granulocyte signaling under GK-high conditions, indicative of enhanced tissue repair and anti-inflammatory signaling that was largely absent in the GK-low condition (**Figure 5d**).

The IGFBP signaling network also underwent pronounced remodeling. Under GK-high conditions, fibroblast-derived signals expanded significantly, extending beyond endothelial cells to additionally target macrophages, T cells, and granulocytes. Conversely, IGFBP signaling remained confined primarily to endothelial cells in the GK-low group, suggesting a link between cuproptosis, metabolic state, and immune response modulation in DFU tissues (**Figure 5e**).

Finally, KIT signaling displayed a notable functional transition: in the GK-low group, signaling primarily originated from endothelial cells, maintaining local homeostasis directed toward fibroblasts and mast cells. In contrast, under GK-high conditions, fibroblasts became predominant signal sources, actively targeting macrophages and mast cells, suggesting a shift in fibroblast function from responsive to regulatory under elevated GK expression (**Figure 5f**).

Collectively, these data are consistent with the hypothesis that elevated GK expression is accompanied by remodeling of cellular communication networks in DFUs, with fibroblasts predicted to exert greater control over key immune-regulatory pathways (MK, HGF, IGFBP, and KIT), thus reinforcing fibroblast-driven immune regulation and tissue remodeling. Together, these results are consistent with the possibility that a cuproptosis-enriched transcriptional context and higher GK expression are accompanied by remodeling of predicted cell–cell communication networks; direct mechanistic links remain to be experimentally validated.

### 3.6 Animal experiments validate elevated GK expression associated with diabetic wounds

Animal experiments confirmed the involvement of GK in diabetic wound tissues through quantitative PCR (qPCR) and Western blot analyses.

**Figure 6:**
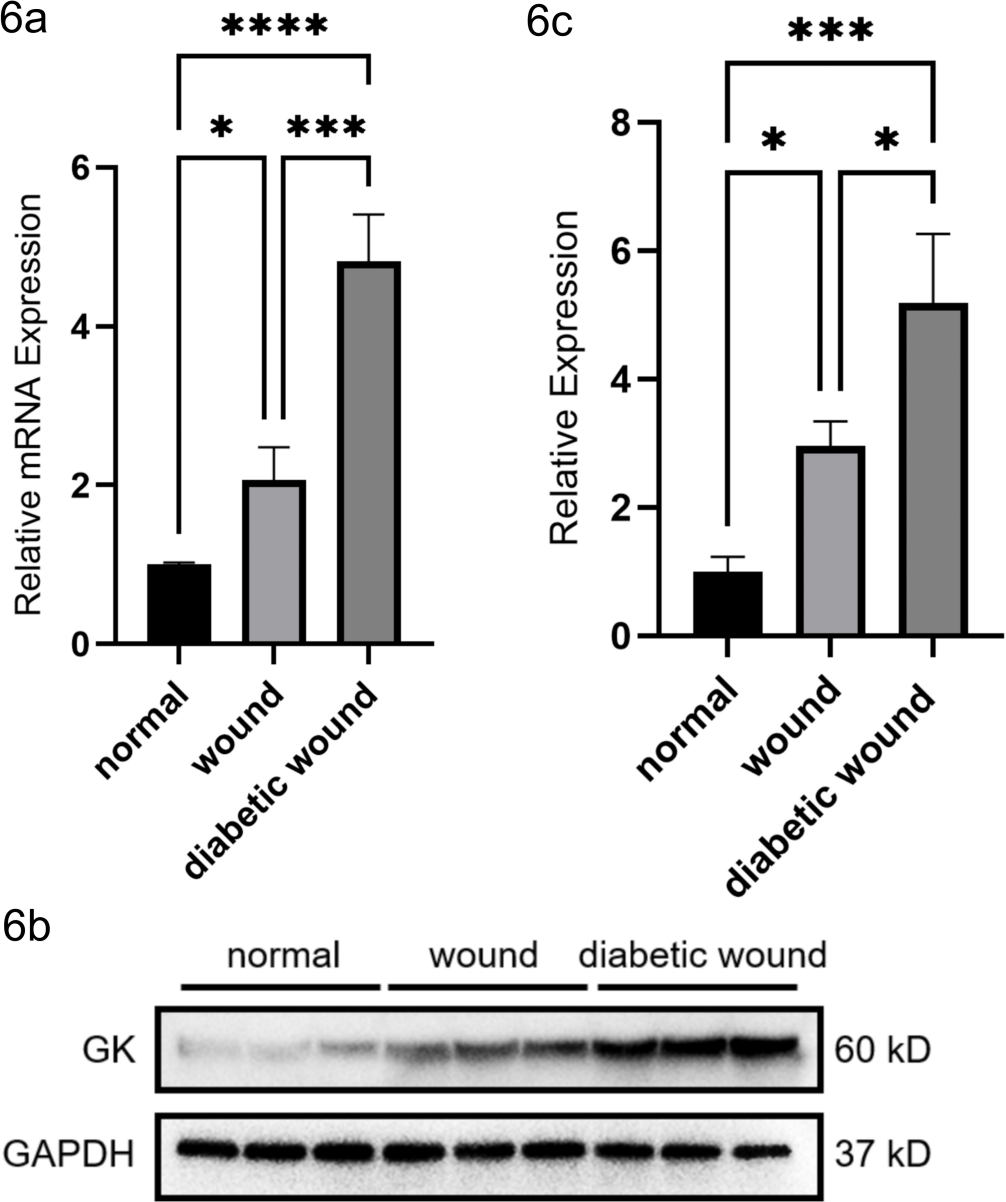
Validation of GK expression in wound and diabetic wound tissues by qPCR and Western blot analysis. **(a)** Relative mRNA expression of GK in normal skin, acute wound tissue, and diabetic wound tissue as determined by quantitative PCR. GK expression was significantly elevated in both the wound group and diabetic wound group compared with the normal group, with the diabetic wound group showing the highest expression level (*p < 0.05, **p < 0.001, ***p < 0.0001, ****p < 0.00001). **(b)** Representative Western blot bands showing GK and GAPDH protein levels in normal, wound, and diabetic wound tissues. Protein lysates were analyzed from each group, confirming the increased expression of GK in diabetic wound tissue. **(c)** Quantification of relative GK protein expression based on Western blot analysis. Band intensities were normalized to GAPDH. A stepwise increase in GK protein expression was observed from normal to wound and diabetic wound groups (*p < 0.05, **p < 0.001, ***p < 0.0001, ****p < 0.00001). Data are presented as mean ± SEM; statistical significance was assessed by one-way ANOVA followed by Tukey’s *post*-*hoc* test.

qPCR results demonstrated that GK mRNA expression was significantly elevated in both wound model and diabetic wound model groups compared with the control group. Notably, the diabetic wound group exhibited substantially higher GK mRNA levels compared with the wound group. Specifically, the GK mRNA expression in the wound model group and diabetic wound model group increased approximately 2.1-fold (p < 0.01) and 3.5-fold (p < 0.001), respectively, compared with the control group. Moreover, GK mRNA expression in the diabetic wound group was significantly higher than that in the wound model group (p < 0.01) (**Figure 6a**).

Western blot analysis revealed a similar trend at the protein level. GK protein expression increased progressively from the control group to the diabetic wound group. The relative GK protein expression increased approximately 2.3-fold (p < 0.01) in the wound group and 4.4-fold (p < 0.001) in the diabetic wound group compared with the control. Additionally, the diabetic wound group exhibited significantly greater GK protein expression compared with the wound model group (p < 0.01) (**Figures 6b**, **6c**).

These animal model results were consistent with the qPCR findings, clearly demonstrating that GK expression, both at the mRNA and protein levels, was significantly elevated in diabetic wound tissues, supporting its potential role in diabetic wound pathology. Collectively, these data confirm that GK is upregulated in diabetic wound tissues, supporting its candidacy as a biomarker; whether GK is a viable therapeutic target requires dedicated functional studies.

## 4 Discussion

In this study, we uncover a novel link between copper‐mediated cell death pathways and the chronic inflammatory state of diabetic foot ulcers (DFUs). Our single-cell analysis identified dermal fibroblasts as the most cuproptosis-enriched cell type in DFU tissue, highlighting these stromal cells as a focal point of copper dysregulation. This finding is intriguing in light of fibroblasts’ dual role as extracellular matrix producers and immunoregulatory “sentinels” in skin repair (15, 16). Cuproptosis, a recently characterized form of regulated cell death triggered by excess intracellular copper, causes proteotoxic stress by aggregating lipoylated mitochondrial enzymes (17). Dysregulated copper homeostasis has been implicated in chronic metabolic and inflammatory diseases (18), but its involvement in non-healing wounds was previously unknown. Our results extend this paradigm by suggesting that DFU fibroblasts experience copper-induced stress or death, which may contribute to their dysfunction in chronic ulcers. In essence, fibroblasts in DFUs appear to be not only victims of a copper-rich microenvironment but also active participants in driving inflammation under these conditions.

Consistent with this idea, we found that fibroblasts in DFUs upregulate a broad suite of immune and inflammatory genes. We identified 543 fibroblast-specific differentially expressed genes (DEGs) in DFU tissue, enriched in pathways such as NF-κB, IL-17, chemokine signaling, and other immune-regulatory cascades. This transcriptional reprogramming suggests that DFU fibroblasts adopt a pro-inflammatory phenotype. Indeed, emerging evidence indicates that a subset of “secretory” or inflammatory fibroblasts arises in chronic non-healing wounds (19). Agrawal et al., for example, described a population of CD40^+^ fibroblasts in DFUs that produces high levels of IL-6, IL-1β, and TNF-α, thereby perpetuating local inflammation (19). Single-cell analyses of healing versus non-healing ulcers have likewise shown that non-healing DFUs harbor fibroblast subsets with heightened expression of injury-response and immune genes (7). Our findings confirm and extend these observations—the DFU fibroblast is not a quiescent bystander but rather an active source of cytokines, chemokines, and danger signals. Through this positive feedback loop of fibroblast–immune activation (15), the wound microenvironment may become locked in a state of persistent inflammation. Prior studies of chronic DFUs have indeed documented an abundance of pro-inflammatory mediators and sustained neutrophil infiltration accompanying impaired healing (20). Thus, the immune pathway enrichment in fibroblasts that we observed provides a cellular basis for the “stalled” inflammatory milieu of DFUs.

A key finding of our study is the identification of GK as a cuproptosis-associated candidate biomarker and possible mechanistic linchpin in DFU pathology. GK (glycerol kinase) was not part of the predefined cuproptosis gene set. However, through subsequent bioinformatics analyses, including differential gene expression and immune cell interaction studies, GK emerged as a key gene associated with the immune microenvironment in diabetic foot ulcers (DFUs). Further validation experiments supported its potential role in the cuproptosis pathway. Using multi-algorithm machine learning, GK emerged as the top fibroblast-derived gene distinguishing DFU tissue (AUC 0.929). GK encodes glycerol kinase, classically known for glycerol metabolism, but our data suggest it has an unrecognized role in regulating fibroblast behavior under stress. Notably, GK expression was strongly correlated with immune cell alterations in DFUs—high GK levels were associated with elevated neutrophil infiltration and reductions in CD8^+^ T cells and T_fh cells. This pattern mirrors the immune dysregulation characteristic of chronic wounds, where an overabundance of neutrophils and a deficit of functional T cells contribute to tissue damage and poor healing (7, 20).

We speculate that GK upregulation may mark a maladaptive fibroblast state that amplifies innate immune recruitment while blunting adaptive immune engagement. Mechanistically, recent work has linked GK activity to the senescence-associated secretory phenotype in fibroblasts [21. In human fibroblasts, glycerol kinase can drive the accumulation of glycerol-3-phosphate and trigger a pro-inflammatory secretome (e.g., IL-6, IL-8) that reinforces chronic inflammation (21). It is tempting to draw a parallel in DFUs: Elevated GK in fibroblasts may induce a senescence-like program—characterized by metabolic derangements and inflammatory secretions—thereby promoting neutrophil-dominated inflammation and impeding T-cell-mediated resolution. Such a scenario is consistent with the chronic, non-resolving inflammation seen in DFUs and raises the hypothesis that GK-marked fibroblast states may contribute to maintaining this inflammatory milieu.

Our cell–cell communication analysis suggests that tissues with higher GK expression are characterized by a rewired signaling network, in which fibroblasts are predicted to act as prominent communication hubs within the DFU microenvironment. Under high GK expression, fibroblasts became dominant hubs of crosstalk, engaging in markedly enhanced interactions with immune cells via several ligand–receptor axes. Notably, we observed increased fibroblast-to-immune signaling through the midkine (MK), hepatocyte growth factor (HGF), IGF-binding protein (IGFBP), and KIT (stem cell factor) pathways in GK-high ulcers. These pathways are each known to modulate inflammation or tissue remodeling. For example, HGF can influence macrophage activation and T-cell functions, and it promotes regenerative wound healing in normal contexts (15). SCF (the KIT ligand) derived from fibroblasts can recruit and activate mast cells, amplifying inflammatory cell influx. Midkine is a stress-induced cytokine that in some contexts acts as an immunomodulator, and IGFBPs can alter cell growth and survival signals in the wound. The net effect of upregulation of these pathways in GK-high conditions is a denser, more immune cell–interactive network centered on fibroblasts. In essence, when GK is high, fibroblasts are predicted to act as more prominent communication hubs in the DFU microenvironment, with enhanced inferred crosstalk with immune cells. Our CellChat analysis indicated that in GK-low conditions, fibroblasts played a relatively minor signaling role (with endothelial cells or other stromal cells mediating many interactions), whereas GK-high conditions shifted the balance such that fibroblasts were sending a much greater share of signals to macrophages, granulocytes, and mast cells. This remodeling of cell communication underscores how a metabolic perturbation (reflected by GK) can instigate widespread changes in intercellular signaling. It also aligns with the concept that fibroblasts are highly plastic and can adopt an “inflammatory” phenotype that orchestrates chronic wound pathology. By prediction, GK-high conditions may enhance multiple pro-inflammatory and pro-fibrotic signaling loops, which could reinforce immune dysregulation; nevertheless, this remains to be directly tested experimentally.

Our *in vivo* experiments lend further credence to the importance of GK. In a diabetic wound rat model, we observed significantly elevated GK mRNA and protein expression in wound-edge tissue, especially under diabetic conditions. GK levels were modestly upregulated in acute wounds of normoglycemic rats but were dramatically higher in chronic diabetic wounds. This *in vivo* confirmation suggests that GK elevation is a bona fide feature of the diabetic wound healing response, not merely an artifact of bioinformatic analysis. It is noteworthy that GK expression increased in parallel with the chronicity of the wound: Diabetic wounds (which heal poorly) showed higher GK than acute wounds in non-diabetic animals. This pattern is consistent with our hypothesis that GK is linked to impaired healing outcomes. It also echoes recent human data—for instance, Yin et al. identified a distinct APOE^+^ fibroblast subset in DFUs that drives fibrosis and inflammation, and they found that high-glucose conditions directly induced a pro-fibrotic, pro-inflammatory phenotype in fibroblasts (22). Our rat model findings, together with such human studies, reinforce the concept that the diabetic milieu (high glucose and associated metabolic stress) can reprogram fibroblasts toward a pathogenic state marked by GK upregulation and enhanced inflammatory signaling. Future studies should explore whether normalizing GK expression or activity can shift fibroblasts back toward a pro-healing state.

Our rat model findings, together with such human studies, reinforce the concept that the diabetic milieu (high glucose and associated metabolic stress) can reprogram fibroblasts toward a pathogenic state marked by GK upregulation and enhanced inflammatory signaling. Given the relatively small sample size of the bulk dataset, we recognize the potential risk of overfitting in our machine learning analysis. To address this, we incorporated cross-validation techniques and external validation through scRNA-seq data and *in vivo* experiments. Future studies involving larger, independent cohorts will be essential to further confirm the diagnostic utility of GK. While our findings suggest a critical involvement of GK in reshaping immune communication in DFUs. The mechanistic roles of GK in cuproptosis and fibroblast-mediated immune signaling require further experimental validation. Meanwhile, the diagnostic performance of GK observed in this study should be considered preliminary. Future research involving external validation in independent datasets will be essential to confirm its diagnostic utility. *In vivo*, we note that the small group size and the lack of functional manipulation of GK significantly limit any mechanistic conclusions. The animal data primarily support an association between diabetic status, wound chronicity, and increased GK expression, rather than establishing a direct causal relationship. These findings should be interpreted with caution, and further studies with larger sample sizes and functional experiments are needed to elucidate the exact role of GK in diabetic wound healing.

Finally, we acknowledge that DFU pathology is multifactorial—neuropathy, ischemia, infection, and other cell types (e.g., keratinocytes and endothelial cells) also play crucial roles. Our focus on fibroblast-centric communication provides one piece of this puzzle. Future research should integrate fibroblast-driven mechanisms with these other aspects to develop a holistic therapeutic strategy. Our study has several important limitations. First, the roles of GK and cuproptosis in DFUs are inferred from integrated transcriptomic analyses and observational animal data and therefore remain correlative. We did not perform direct functional manipulation of GK (e.g., siRNA/shRNA knockdown and overexpression) or perturb copper homeostasis (e.g., copper chelators or ionophores) in fibroblasts or immune cells, nor did we measure canonical markers of cuproptosis. As a result, we cannot conclude that GK causally regulates cuproptosis or immune-cell behavior in DFUs. Future mechanistic studies using targeted GK gain- and loss-of-function approaches, combined with *in vitro* and *in vivo* assays of cuproptosis and immune responses in fibroblasts and immune cell subsets, will be essential to establish causality and to evaluate whether GK is a viable therapeutic target.

In summary, our study identifies GK and cuproptosis-related signaling as promising new leads in the quest to understand and ultimately break the cycle of chronic inflammation in diabetic foot ulcers. Continued investigation along these lines, with rigorous *in vivo* validation and translational studies, is warranted to translate these insights into improved outcomes for patients with DFUs.

## Data Availability

The original contributions presented in the study are included in the article/**Supplementary Material**. Further inquiries can be directed to the corresponding author.

## Supplementary material

Supplementary Table 1 Numbers of cells retained per sample and per major cell type after quality control and annotation (GSE231643).
