# Supplementary material for: Cuproptosis-driven reprogramming of fibroblast communication by GK is associated with the immune microenvironment in diabetic foot ulcers
Source: Front Immunol. 2026 Jan 9;16:1687806. doi: 10.3389/fimmu.2025.1687806 (PMC12827545; doi:10.3389/fimmu.2025.1687806)
Supplement: Supplementary Table 1 — Numbers of cells retained per sample and per major cell type after quality control and annotation (GSE231643). [file Table1.docx]

Table S1. Numbers of Cells Retained per Sample and per Major Cell Type After Quality Control and Annotation (GSE231643)

| Group | Sample | Cells retained after QC (n) | Percent of retained cells (%) |
| --- | --- | --- | --- |
| DFU | DFU1 | 1248 | 4.95 |
| DFU | DFU2 | 2309 | 9.16 |
| DFU | DFU3 | 2840 | 11.27 |
| Healthy | Healthy1 | 4701 | 18.66 |
| Healthy | Healthy2 | 4790 | 19.01 |
| Healthy | Healthy3 | 2075 | 8.23 |
| Healthy | Healthy4 | 6036 | 23.95 |
| Healthy | Healthy5 | 1199 | 4.76 |
| Total | | 25198 | 100 |

| Major Cell Types (After QC + Annotation) | | |
| --- | --- | --- |
| Major cell type | Cells retained (n) | Notes |
| Macrophages | 5,888 | Fill with post-QC, post-annotation counts |
| Fibroblasts | 8,388 | Fill with post-QC, post-annotation counts |
| T cells | 4,291 | Fill with post-QC, post-annotation counts |
| B cells | 1,147 | Fill with post-QC, post-annotation counts |
| Dendritic cells | 405 | Fill with post-QC, post-annotation counts |
| Endothelial cells | 3,814 | Fill with post-QC, post-annotation counts |
| Granulocytes | 1,079 | Fill with post-QC, post-annotation counts |
| Mast cells | 186 | Fill with post-QC, post-annotation counts |
